# Parallels between spacing effects during behavioral and cellular learning

**DOI:** 10.3389/fnhum.2012.00203

**Published:** 2012-07-06

**Authors:** Jürgen Kornmeier, Zrinka Sosic-Vasic

**Affiliations:** ^1^Institute for Frontier Areas of Psychology and Mental HealthFreiburg, Germany; ^2^University Eye-HospitalFreiburg, Germany; ^3^Transfercenter for Neuroscience and Learning, University of UlmUlm, Germany; ^4^Department of Psychiatry and Psychotherapy, University Clinic of UlmUlm, Germany

**Keywords:** spacing effect, memory, learning, synaptic plasticity, long term potentiation (LTP)

## Abstract

Repeated learning improves memory. Temporally distributed (“spaced”) learning can be twice as efficient than massed learning. Importantly, learning success is a non-monotonic maximum function of the spacing interval between learning units. Further optimal spacing intervals seem to exist at different time scales from seconds to days. We briefly review the current state of knowledge about this “spacing effect” and then discuss very similar but so far little noticed spacing patterns during a form of synaptic plasticity at the cellular level, called long term potentiation (LTP). The optimization of learning is highly relevant for all of us. It may be realized easily with appropriate spacing. In our view, the generality of the spacing effect points to basic mechanisms worth for coordinated research on the different levels of complexity.

## The spacing effect

Our ability to store information and to use it in a reflexive way is essential for the successful interaction with our environment. Understanding the mechanisms underlying learning and memory is a central goal in cognitive and neurosciences. A promising strategy in this direction may be to study parameters that optimize learning and memory.

Increasing the number of learning repetitions (e.g., memorizing vocabularies more often) improves memory, which is well known. Less well known may be, however, that massed learning is less efficient than spaced learning: two spaced learning sessions can be twice as efficient as two learning sessions without spacing (“massed” sessions). This means that the same amount of learning time can be twice as efficient if executed with the optimal spacing schedule. This phenomenon is called the “spacing effect”. As early as 1885 the German psychologist Herrmann Ebbinghaus observed in one of his numerous memory experiments that 38 learning repetitions distributed over three days resulted in the same memory performance as with 68 massed repetitions (Ebbinghaus, 1913). “Spacing interval” can mean the time without mental engagement between two presentations of the learning content. In principle, the learner can use this time to rehearse the learning content mentally. Alternatively, the spacing interval can be filled with different learning contents and quantified by their number.

### Generality of the spacing effect

Spacing effects have been found with a variety of test paradigms including free recall, cued recall, and recognition memory (however see Litman and Davachi, 2010), and for a multitude of learning materials, like sense and nonsense syllables, words, word pairs (e.g., vocabulary learning), pictures, arithmetic rules, scientific, and mathematical concepts, or scientific terms (e.g., Ebbinghaus, 1913; Dempster, 1996). Spacing effects occur with multimodal (auditory and visual) stimulation (Janiszewski et al., 2003), with intentional, and also with incidental learning (e.g., Challis, 1993; Toppino et al., 2002). The spacing effect is observed from 4-year-old children (Rea and Modigliani, 1987) up to 76 year old seniors (Balota et al., 1989; Toppino, 1991). It has been also observed in animals such as rodents (Lattal, 1999), Aplysia (Mauelshagen et al., 1998), and even within Drosophila (Yin et al., 1995). Due to this universality, the mechanisms underlying the spacing effect are assumed to be basic and highly automatic (Rea and Modigliani, 1987; Toppino, 1991; Dempster, 1996).

### Learning depends on the spacing interval duration

**Figure 1:**
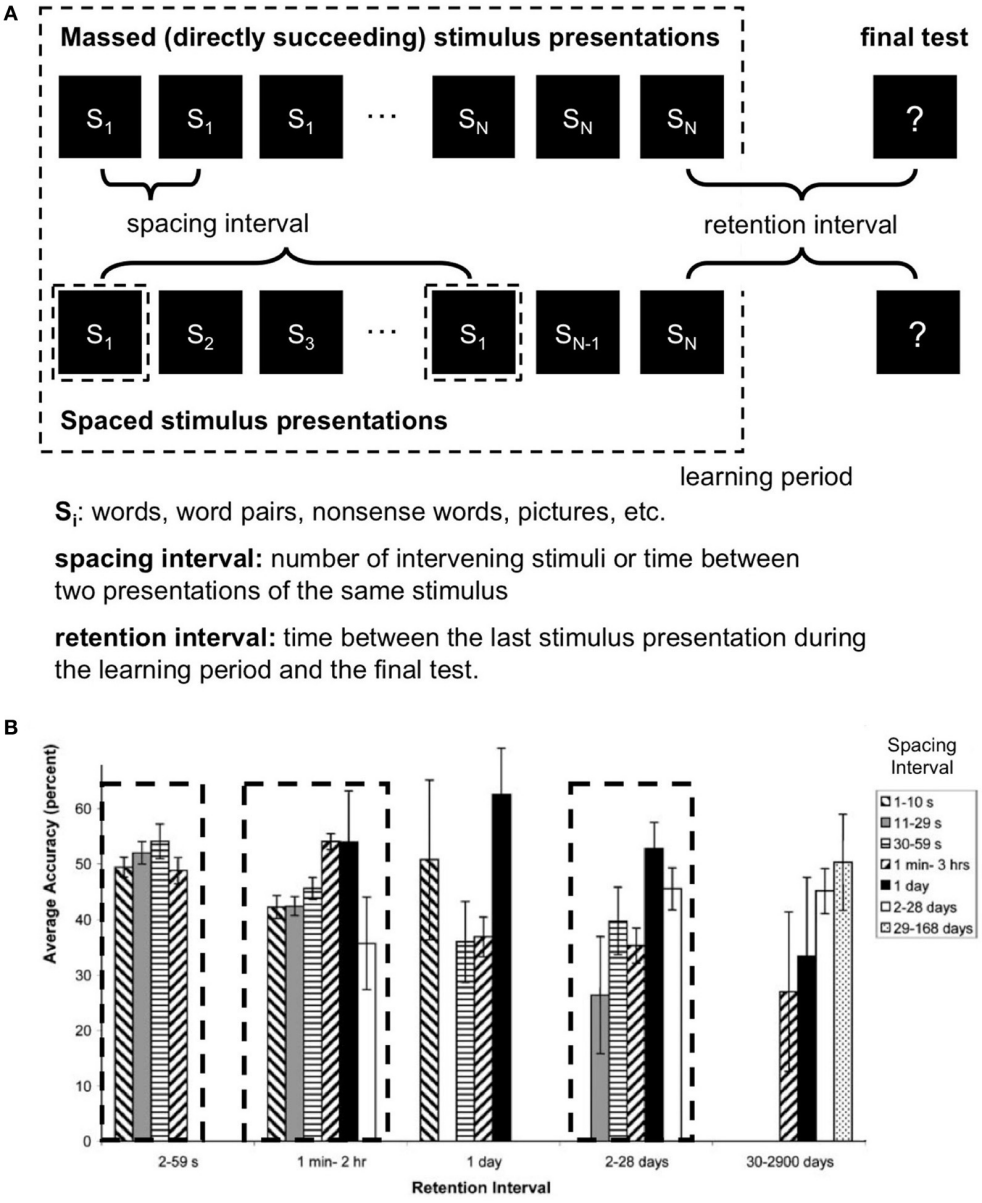
**(A)** Typical spacing paradigm. **(B)** Spacing effects at different time scales. Average values from 187 studies (after Cepeda et al., 2006, Figure 6, modified). Local maxima of memory performance (accuracy) with certain combinations of spacing and retention intervals are indicated by dashed rectangles. Error bars represent one standard error of the mean. s = seconds; min = minutes; hr = hour.

Increasing the spacing interval, i.e., the time between learning repetitions (Figure 1A, Melton, 1970; Toppino and Bloom, 2002; Kahana and Howard, 2005) improves memory performance. However, it turns out that this effect is non-monotonic: After a certain optimal spacing interval, memory performance declines again (Donovan and Radosevich, 1999; Toppino and Bloom, 2002; Cepeda et al., 2009).

### Learning depends on the retention interval duration

An also important but rather little noted finding comes from Cepeda et al.'s meta-analysis (2006). To date, most of the studies about spacing effects compared different spacing intervals but kept the time between the last learning unit and a final test (“retention interval,” Figure 1A) constant. Comparisons between studies, however, indicate interdependence between the spacing interval and the retention interval: longer retention intervals are coupled with longer optimal spacing intervals (Bahrick and Phelps, 1987; Dempster, 1987).

Of note, another so far little noted finding is the wide range of spacing intervals from seconds to months (Cepeda et al., 2006). As Cepeda et al. (2006) nicely summarized, there is an interaction between spacing interval and retention interval, suggesting more than one optimal pair of spacing—and retention-values. Thus, a 3D-representation of the dimensions spacing interval, retention interval and memory performance seems to contain several maxima at different time scales (Figure 1B for a 2D visualization of the 3D parameter space).

## Spacing effect meets synaptic plasticity

Most models for the spacing effect are exclusively cognitive without addressing neurobiological aspects (for overviews see Dempster, 1996; Cepeda et al., 2006). In the present opinion paper we want to direct attention to significant parallels between findings at the cognitive level about spacing effects and related findings at the cellular level with synaptic plasticity.

There is a common agreement that learning and memory are neurally implemented by synaptic plasticity, i.e., a change of synaptic strength, resulting in an enhancement or a reduction of signal transmission between two neurons. Two most often discussed mechanisms of synaptic plasticity are long-term potentiation (LTP) and long-term depression (LTD).

LTD is a reduction of synaptic efficacy lasting for hours or longer. It can be induced either by transient strong or persistent weak synaptic stimulations. In protocols with repetitive paired pre- and postsynaptic stimulation the induction of LTD or its opposite LTP (see below) strongly depends on the stimulation frequency and on the order of pre- and postsynaptic stimulation (e.g., Dan and Poo, 2006).

LTP is an enhancement of a chemical synapse between two neurons, lasting from minutes to months or longer. It is typically induced by high frequency stimulation (“HFS”) or by stimulation protocols with simultaneous pre- and postsynaptic neural stimulation (e.g., Cooke and Bliss, 2006). After LTP induction less presynaptic activity is necessary to induce postsynaptic action potentials than before, i.e., the flow of information via this specific synapse is facilitated significantly.

Several features qualify LTP as a widely discussed cellular mechanism for learning and memory:

1. Input specificity. The transmission improvement is restricted to specific synapses. Thus, a single pathway can be potentiated without affecting neighboring pathways.
2. Associativity. A “weak” stimulus can be potentiated through association with a strong stimulus. Alternatively, several weak stimulations of pathways that converge on a small area of a postsynaptic membrane sum up. This may be labeled as a simple form of associative learning.
3. There are several patterns of correlation between learning and memory on a behavioral level and LTP on a cellular level. Especially several chemical agents can enhance or inhibit plasticity at both levels (Cooke and Bliss, 2006).

LTP has been found in animal cell slices (e.g., Lynch, 2003), in nearly all areas of the living mammalian brain (Lynch, 2004), including humans and human cell slices (Cooke and Bliss, 2006). LTP is found in different areas of the brain, like amygdala (Clugnet and LeDoux, 1990), hippocampus, cerebellum, and cortex (e.g., Malenka and Bear, 2004). Detailed LTP mechanisms differ between brain areas in a number of factors, like the contributing neurotransmitters, the intracellular molecular mechanisms and/or contributing membrane receptors, etc. (Malenka and Bear, 2004).

Three major LTP phases have been distinguished (e.g., Abraham, 2003):

LTP 1 has decay times in the order of hours. It is independent of protein synthesis and relies on phosphorylation of synaptic receptors, on the variation of their number (e.g., Malenka and Bear, 2004), and on the alternation of intrinsic properties of their ligand-gated ion channels.

LTP 2 has a decay time in the order of days. It seems to rely on new syntheses (translation) of dendritic proteins from pre-existing messenger RNA.

LTP 3 has a decay time in the order of months. It relies on altered gene transcription and production of new proteins. Structural changes to the shape of existing synapses and the generation of new synapses were observed.

Most important for any type of stimulation protocol is, that a certain level of postsynaptic depolarisation (excitatory postsynaptic polarisation, “EPSP”) has to be reached for LTP induction. Which LTP phase will be attained depends on the stimulation protocol. Typically, HFS protocols were used: Trains of 50–100 electrical pulses with a typical frequency of 100 Hz for a certain time interval (e.g., 1 s) are applied repetitive with spacing intervals without stimulation in between. Spacing intervals differ between studies (from 0.5 s to 10 min or even longer, e.g., Albensi et al., 2007).

Standard HFS protocols, however, are highly artificial and somewhat unphysiological, although recent studies indicate visual perceptual learning with typical LTP and LTD protocols (Normann et al., 2007; Beste et al., 2011; Aberg and Herzog, 2012). Interestingly, there is accumulating evidence for a dominating theta-to-alpha rhythm (5–12 Hz) in both the EEG (e.g., Grastyan et al., 1959) and the high-frequency hippocampal discharge bursts (e.g., Graves et al., 1990) of learning animals. Several studies even found more efficient LTP induction with more physiological theta/alpha stimulation train protocols compared to HFS protocols (Albensi et al., 2007). Further, trains of transcranial magnetic stimulation (TMS) in this frequency range can induce long-lasting changes in the human motor cortical output (Huang et al., 2005; Cooke and Bliss, 2006). Thus LTP can also be induced under physiological conditions.

In summary, the number of pulses, their frequency, the number of stimulation units, their duration and the duration of the spacing intervals between units can differ between studies and seem to be differentially efficient in both cellular LTP and behavioral learning. A number of similarities between LTP and learning on the behavioral level point to a possible aetiological association and are listed in Table 1.

**Table 1:** **Parallels between Spacing-Effects and LTP**.

**LTP**	**Spacing-Effect**
**ADVANTAGE OF SPACED COMPARED TO MASSED STIMULATION/LEARNING**	
The duration of LTP strongly depends on the stimulation protocol. Protocols with temporal spaced stimulation trains lead to longer lasting LTP than protocols with massed stimulation (Huang and Kandel, 1994; Abraham et al., 2002; Scharf et al., 2002; Zhou et al., 2003). The longest lasting LTP had been induced by stimulation trains distributed over days (Racine et al., 1995).	The memory performance strongly depends on the learning protocol: Protocols with temporal spaced learning units lead to better memory performance (up to 250%) than protocols with massed learning units (Bahrick and Phelps, 1987; Dempster, 1987). Efficient Spacing Intervals are in the span of seconds to weeks (Cepeda et al., 2006; Roediger and Karpicke, 2006).
**LEARNING AS A NON-MONOTONIC FUNCTION OF THE SPACING INTERVAL**	
LTP enhancement is a non-monotonic peak function of the stimulation protocol (Albensi et al., 2007), and especially of the spacing interval between stimulations.	Behavioral memory performance is a non-monotonic peak function of the spacing interval between learning units (Donovan and Radosevich, 1999; Toppino and Bloom, 2002; Cepeda et al., 2009).
**SPACING EFFECTS OCCUR ON DIFFERENT TIME SCALES**	
LTP is induced in phases (LTP 1-3) with increasing time constants for memory performance (e.g., Lynch, 2004; Raymond, 2007). Which LTP phase is reached depends on the stimulation protocol (e.g., Abraham, 2003; Albensi et al., 2007).	Learning seems to take place in phases with optimal spacing intervals for maximal memory performance. Which phase is reached depends on the magnitude of the Spacing-Interval (Cepeda et al., 2006, see Figure 1B).

**Some Outstanding Questions**

**(1) What are the Optimal Spacing Intervals?**

Only few stimulation protocols for LTP induction have been used so far (e.g., Albensi et al., 2007), the major part of the parameter space is unexplored. Especially, very little is known about optimal spacing of the stimulations to induce LTP. In addition, more knowledge about the parameter space of behavioral spacing effects and optimal spacing intervals is needed (Cepeda et al., 2006). Identifying spacing time constants at both levels of complexity and comparing them may be a fruitful approach on the way to better understand the relation between low- and high-level plasticity.

**(2) Memory Capacity or Memory Lifetime?**

LTP studies typically report the duration of LTP as a measure of learning and memory (e.g., Abraham, 2003). Behavioral spacing studies typically measure memory performance as the number of memorized items at one or two certain time points after learning. The duration of behavioral spacing effects has not been systematically studied, but is necessary to understand the relation between low- and high-level spacing effects.

**(3) Spaced LTP Induction vs. Spaced Behavioral Learning**

One typical pyramidal neuron has roughly 8.000 afferent and between 6.000 and 24.000 efferent synapses to other neurons (Braitenberg and Schütz, 1998). How can principles from LTP at single synapses be related to learning on the behavioral level, based on a highly complex interaction of a network of thousands to millions of neurons? Several studies with awake animals and humans indicate stimulation and learning patterns and dependencies very similar to those known from LTP (e.g., Malenka and Bear, 2004; Cooke and Bliss, 2006; Dan and Poo, 2006; Beste et al., 2011; Aberg and Herzog, 2012) although whole network and not single neurons were stimulated. Thus patterns of neural plasticity known from individual synapses seem to occur at higher levels as well.

We agree with Dan and Poo (Dan and Poo, 2006) that simple and analytically tractable phenomenological rules may sometimes be a good starting point to understand mechanisms on a more complex level. It is thus worthwhile to look for such rules by comparing spacing-dependent plasticity at different levels of complexity. An interesting approach to bridge the gap has recently proposed by Aberg and Herzog (Aberg and Herzog, 2012).

## Conclusions

Successful learning depends on several factors. Highly important are (1) the number of learning units and (2) the temporal distance (spacing intervals) between learning units (amongst other factors). The universality of the behavioral spacing effects and the parallels to spacing effects at the LTP level indicate that basic learning mechanisms are at work. Clearly LTP takes place at the level of synapses and molecules, whereas most findings form the spacing literature are from a complex behavioral/system level. Further, we do not know whether LTP induced by spaced stimulations directly contributes to the improved long-term memory seen after spaced studying at the behavioral level. However, the patterns described above suggest an intriguing correlation between synaptic and cognitive processes, worth to be investigated further. Comparison of optimal spacing values for learning tasks from different levels of cognition with optimal spacing intervals between LTP stimulation units may further elucidate the interrelation between low and high level learning in a systematic manner. Related results may help to deepen our understanding of basic learning mechanisms on one hand and provide simple, efficient, and universally applicable learning rules on the other hand.

### Conflict of interest statement

The authors declare that the research was conducted in the absence of any commercial or financial relationships that could be construed as a potential conflict of interest.
